# Risk factors for community-associated *Clostridioides difficile* infection in young children

**DOI:** 10.1017/S0950268819000372

**Published:** 2019-04-05

**Authors:** M. K. Weng, S. H. Adkins, W. Bamberg, M. M. Farley, C. C. Espinosa, L. Wilson, R. Perlmutter, S. Holzbauer, T. Whitten, E. C. Phipps, E. B. Hancock, G. Dumyati, D. S. Nelson, Z. G. Beldavs, V. Ocampo, C. M. Davis, B. Rue, L. Korhonen, L. C. McDonald, A. Y. Guh

**Affiliations:** 1 Division of Healthcare Quality Promotion, Centers for Disease Control and Prevention, Atlanta, GA, USA; 2 Colorado Department of Public Health and Environment, Denver, CO, USA; 3 Department of Medicine, Emory University, Atlanta, GA, USA; 4 Georgia Emerging Infections Program, Decatur, GA, USA; 5 Atlanta Veterans Affairs Medical Center, Atlanta, GA, USA; 6 Maryland Department of Health, Baltimore, MD, USA; 7 Minnesota Department of Health, St Paul, MN, USA; 8 Career Epidemiology Field Officer Program, Centers for Disease Control and Prevention, Atlanta, GA, USA; 9 New Mexico Emerging Infections Program, University of New Mexico, Albuquerque, NM, USA; 10 New York Emerging Infections Program and University of Rochester Medical Center, Rochester, NY, USA; 11 Oregon Health Authority, Portland, OR, USA; 12 Tennessee Department of Health, Nashville, TN, USA

**Keywords:** *Clostridium difficile*

## Abstract

The majority of paediatric *Clostridioides difficile* infections (CDI) are community-associated (CA), but few data exist regarding associated risk factors. We conducted a case–control study to evaluate CA-CDI risk factors in young children. Participants were enrolled from eight US sites during October 2014–February 2016. Case-patients were defined as children aged 1–5 years with a positive *C. difficile* specimen collected as an outpatient or ⩽3 days of hospital admission, who had no healthcare facility admission in the prior 12 weeks and no history of CDI. Each case-patient was matched to one control. Caregivers were interviewed regarding relevant exposures. Multivariable conditional logistic regression was performed. Of 68 pairs, 44.1% were female. More case-patients than controls had a comorbidity (33.3% *vs.* 12.1%; *P* = 0.01); recent higher-risk outpatient exposures (34.9% *vs.* 17.7%; *P* = 0.03); recent antibiotic use (54.4% *vs.* 19.4%; *P* < 0.0001); or recent exposure to a household member with diarrhoea (41.3% *vs.* 21.5%; *P* = 0.04). In multivariable analysis, antibiotic exposure in the preceding 12 weeks was significantly associated with CA-CDI (adjusted matched odds ratio, 6.25; 95% CI 2.18–17.96). Improved antibiotic prescribing might reduce CA-CDI in this population. Further evaluation of the potential role of outpatient healthcare and household exposures in *C. difficile* transmission is needed.

## Background

*Clostridioides difficile* (formerly *Clostridium difficile*), a Gram-positive, spore-forming anaerobic bacillus, is the most common cause of healthcare-associated diarrhoea in the USA [1]. Acquisition of *C. difficile*, most frequently through faecal–oral transmission, can lead to asymptomatic colonisation or a range of clinical manifestations from mild diarrhoea to pseudomembranous colitis, bowel perforation or death [2, 3]. Children have not been thought to be a particularly high-risk population for *C. difficile* infection (CDI). However, the severity and incidence of CDI-related hospitalisations have increased in both paediatric and adult populations [4–6], and CDI-related hospitalisations are associated with higher costs and longer length of stay [7–10]. Asymptomatic colonisation with *C. difficile* occurs at much higher rates in infants aged <1 year than in adults [11] but decreases rapidly after the first year of life [12]. In one paediatric study, CDI incidence was found to be highest in those aged 1–3 years, with similar clinical presentation, disease severity and outcomes as older children; coinfection with other enteric pathogens was rare, supporting *C. difficile* as the causative aetiology in this young age group [13].

Although traditionally a healthcare-associated infection, CDI is increasingly spread through community acquisition [14–16]. Among paediatric CDI cases identified through population-based surveillance, 71–75% were determined to be community-associated (CA) [13, 17]. While several studies have assessed CA-CDI risk factors in adults, limited data exist for children. The few studies to examine CA-CDI risk factors in children have primarily focused on traditional risk factors, such as outpatient healthcare and medication exposures [18, 19], although one study also assessed exposures to household members younger than 1 year of age or who had a diagnosis of CDI [18]. The objective of this study was to evaluate various potential healthcare- and community-related risk factors for CA-CDI in children, including different types of outpatient healthcare and household exposures as well as dietary and daycare exposures. Because of the higher incidence of CDI observed in younger children [13], we limited our study to children aged 1–5 years.

## Methods

Active, population-based CDI surveillance is conducted by the US Centers for Disease Control and Prevention (CDC)’s Emerging Infections Program (EIP) in 10 geographically-diverse US sites. During October 2014–February 2016, children residing in the surveillance catchment areas of eight of the 10 EIP sites were enrolled for this study (Colorado, Georgia, Maryland, Minnesota, New Mexico, New York, Oregon and Tennessee), which comprised a surveillance population of >10 million persons. The study protocol was approved by institutional review boards at the CDC and participating EIP sites. Verbal consent from participants’ caregivers was obtained prior to enrolment.

### Case definition and enrolment

All laboratories serving the residents of the EIP surveillance catchment areas reported all positive *C. difficile* test results obtained during routine clinical care to the EIP site staff. For EIP surveillance, a CDI case was defined as a positive *C. difficile* toxin or molecular assay in a person aged 12 months or older who had no positive test in the prior 8 weeks. Based on review of medical records, all cases reported to EIP were classified as either healthcare-associated CDI or community-associated CDI, which was defined as a *C. difficile*-positive stool collected as an outpatient or within 3 days of hospitalisation in a person who had no admission to a healthcare facility in the prior 12 weeks. The definition of healthcare-associated CDI has been described elsewhere [16]. We used the EIP CDI surveillance system to identify community-associated case-patients aged 12–60 months for potential enrolment in the study. Because the EIP CDI case definition was based on a laboratory diagnosis, to increase the likelihood that enrolled case-patients had a true infection and were not colonised with *C. difficile*, only case-patients with a diarrhoeal illness (⩾3 watery stools in a 24 h period) associated with their positive stool specimen were included in the study. Case-patients were interviewed to determine their eligibility for the study. Case-patients who did not report an associated diarrhoeal illness, who did not reside in the EIP surveillance catchment area, who reported an inpatient admission in the prior 12 weeks, or who reported any prior history of CDI or if they had a prior positive *C. difficile* test reported to EIP were considered ineligible for the study. In addition, case-patients were excluded from the study if their caregiver could not be interviewed about their exposures or if they could not be matched to a control within 90 days of the specimen collection date.

### Control enrolment

Each case-patient was matched to one control by site and age group (12–23, 24–47, 48–60 months). Controls were chosen randomly from a commercial database of residential telephone numbers or from birth registries if the subjects were aged 12–23 months. Controls had to reside in the same surveillance catchment area as the matched case-patient at the time of the case-patient's specimen collection. Controls were excluded if they had a diarrhoeal illness or an overnight stay in a healthcare facility within 12 weeks prior to the matched case-patient's onset of illness, or if they ever had a CDI diagnosis.

### Data collection

Trained interviewers used a standardised questionnaire to collect information by telephone. Demographics and underlying comorbidities were recorded. Caregivers were interviewed about participants’ relevant healthcare, household and dietary exposures, as well as water sources within the 12 weeks prior to the case-patient's illness onset date. Medication use was assessed in the 2, 2–4 and 4–12 weeks prior to the case-patient's illness onset date. Additional information about case-patients’ clinical course was collected as part of routine surveillance.

### Statistical analysis

Descriptive analyses were performed to summarise demographic and clinical characteristics. Because there was a low frequency of exposure to several of the outpatient settings, a new variable was created that combined the outpatient exposures into either a higher- or lower-risk exposure category based on criteria used in prior studies [20, 21]. The following outpatient settings were classified as higher-risk exposures: emergency room, outpatient procedure centre, haemodialysis facility, hospital-based outpatient setting, urgent care and ambulatory surgical centre. Lower-risk exposures included the following: dental office, doctor's office, outpatient laboratory and physical therapy centre. Univariate exact conditional logistic regression was performed. Variables with *P*-value <0.20 on the univariate test were entered into a multivariable conditional logistic regression model using stepwise selection to identify CA-CDI predictors. If specific types of outpatient exposures as well as the combined variable of higher- or lower-risk outpatient exposure all had a *P*-value <0.20 on the univariate test, only the combined higher- or lower-risk exposure variable was included in the multivariable model to avoid collinearity. In multivariable analysis, *P-*values <0.05 were considered significant. SAS statistical software version 9.3 (SAS Institute Inc, Cary, NC, USA) was used for the analysis.

## Results

Of the 136 children (68 matched pairs) enrolled in the study, 44.1% were female and 69.1% were 12–23 months old (Table 1). The median number of participants (case-patients and controls) per EIP site was 14 (range: 2–60), with 44.1% of all participants from one of the eight sites, Georgia. The distribution of Georgia and non-Georgia participants did not differ by sex (46.7% *vs.* 42.1% females; *P* = 0.59) and age group (70.0% *vs.* 68.4% were aged 12–23 months; *P* = 0.84).

**Table 1.** Age, sex and state of residence of study participants

Variable	Number of study participants, *n* (%)
Age group	
12–23 months	94 (69.1)
24–47 months	22 (16.2)
48–60 months	20 (14.7)
Sex	
Female	60 (44.1)
State of residence	
Colorado	16 (11.8)
Georgia	60 (44.1)
Maryland	6 (4.4)
Minnesota	14 (10.3)
New Mexico	14 (10.3)
New York	14 (10.3)
Oregon	2 (1.5)
Tennessee	10 (7.4)

Of the 68 case-patients, 64 (94.1%) caregivers interviewed recalled the onset date of diarrhoeal illness. Other commonly reported symptoms included vomiting (58.8%), fever (54.4%) and abdominal pain (50.0%). *C. difficile* diagnostic testing information was available for all 68 case-patients: 22 (32.4%) were positive by a toxin enzyme immunoassay, 10 (14.7%) were positive by a molecular assay only (toxin-negative) and 36 (52.9%) were diagnosed by a laboratory that only utilised a molecular assay (no information available on toxin positivity for these 36 patients). Of the 68 case-patients, only 10 (14.7%) had another enteric pathogen detected at the time of their CDI diagnosis based on either what was documented in the medical record (collected as part of routine surveillance activity) or what the case-patient reported during their interview. For the remaining 58 cases, 48 (82.8%) did not test positive for another enteric pathogen based on medical-record review, and 10 (17.2%) were not tested for another enteric pathogen. Twelve (17.6%) of 68 case-patients required hospitalisation for CDI, but none required admission to the intensive care unit or developed toxic megacolon. CDI treatment information was available for 62 case-patients; of these, 50 (80.6%) received treatment for CDI and 12 (19.4%) did not.

A significantly higher proportion of case-patients than controls had an underlying chronic medical condition (33.3% *vs.* 12.1%; *P* = 0.01), such as congenital heart disease, cystic fibrosis, gastrointestinal disease, asthma, bronchopulmonary dysplasia, neurologic illness or history of organ transplant (Table 2). Neonatal intensive care unit (NICU) stay at time of birth (26.9% *vs.* 11.9%; *P* = 0.04) was also more common in case-patients compared with controls.

**Table 2.** Univariate analysis: select demographic and clinical characteristics and healthcare and medication exposures among study participants

Variable	Cases No. (%)	Controls No. (%)	Unadjusted matched odds ratio (95% CI)	*P-*value
Demographic information				
White race	54/68 (79.4)	46/68 (67.6)	1.73 (0.78–4.01)	0.20
Hispanic/Latino	19/68 (27.9)	18/68 (26.5)	1.08 (0.46–2.59)	1.00
Have health insurance	67/68 (98.5)	67/67 (100.0)	1.00 (0.00–19.00)	1.00
Household income >$50k	37/62 (59.7)	40/64 (62.5)	1.17 (0.50–2.76)	0.85
Select medical conditions				
Any medical condition	22/66 (33.3)	8/66 (12.1)	3.17 (1.22–9.69)	0.01
Congenital heart disease	3/67 (4.5)	1/67 (1.5)	3.00 (0.24–157.49)	0.63
Cystic fibrosis	1/67 (1.5)	0/67 (0.0)	1.00 (0.05–undef)	1.00
Gastrointestinal condition	5/67 (7.5)	1/67 (1.5)	5.00 (0.56–236.49)	0.22
Short-gut disease	3/67 (4.5)	0/67 (0.0)	3.85 (0.58–undef)	0.25
Asthma	3/67 (4.5)	1/66 (1.5)	3.00 (0.24–157.49)	0.63
Bronchopulmonary dysplasia	2/66 (3.0)	0/67 (0.0)	2.41 (0.29–undef)	0.50
Organ transplant	1/67 (1.5)	0/67 (0.0)	1.00 (0.05–undef)	1.00
Neurologic illness	7/67 (10.4)	3/67 (4.5)	2.33 (0.53–13.98)	0.34
Healthcare exposures^a^				
Any higher-risk outpatient exposure^b^	23/66 (34.9)	12/68 (17.7)	4.20 (1.09–19.36)	0.03
Any lower-risk outpatient exposure^b^	31/66 (47.0)	38/68 (55.9)	1.34 (0.50–3.83)	0.68
Exposure to any outpatient setting	54/66 (81.8)	50/68 (73.5)	1.63 (0.62–4.52)	0.38
Dentist's office	8/65 (12.3)	10/68 (14.7)	0.75 (0.21–2.46)	0.79
Doctor's office	47/62 (75.8)	42/68 (61.8)	1.90 (0.84–4.57)	0.14
Emergency department	12/65 (18.5)	5/68 (7.4)	5.00 (1.07–46.93)	0.04
Haemodialysis	0/66 (0.0)	0/68 (0.0)	–	–
Hospital-based outpatient setting	6/66 (9.1)	1/68 (1.5)	6.73 (1.22–undef)	0.06
Outpatient laboratory	1/65 (1.5)	5/68 (7.4)	0.20 (0.004–1.79)	0.22
Outpatient procedure centre	4/65 (6.2)	4/68 (5.9)	1.00 (0.13–7.47)	1.00
Outpatient surgery centre	5/65 (7.7)	0/68 (0.0)	6.73 (1.22–undef)	0.06
Physical therapy centre	4/65 (6.2)	1/68 (1.5)	4.00 (0.39–196.99)	0.38
Urgent care	3/65 (4.6)	3/68 (4.4)	1.00 (0.13–7.47)	1.00
Received any outpatient procedure	12/65 (18.5)	13/68 (19.1)	0.89 (0.29–2.59)	1.00
Dental cleaning	6/65 (9.2)	10/68 (14.7)	0.50 (0.11–1.87)	0.39
Endoscopy	1/65 (1.5)	1/68 (1.5)	1.00 (0.01–78.49)	1.00
X-ray that required bowel preparation	0/65 (0.0)	1/68 (1.5)	1.00 (0.00–19.00)	1.00
Surgical procedure	6/65 (9.2)	3/68 (4.4)	2.00 (0.43–12.36)	0.51
Visited or accompanied a person to a healthcare facility	18/66 (27.3)	12/67 (17.9)	1.50 (0.63–3.73)	0.42
Had a feeding tube	2/68 (2.9)	0/68 (0.0)	2.41 (0.29–undef)	0.50
Birth				
Delivered via C-section	19/67 (28.4)	22/67 (32.8)	0.84 (0.41–1.73)	0.74
Stayed in NICU	18/67 (26.9)	8/67 (11.9)	3.00 (1.04–10.55)	0.04
Medication exposures^a^				
Any antibiotic use in prior 12 weeks	37/68 (54.4)	13/67 (19.4)	5.80 (2.22–19.19)	<0.0001
Any antibiotic use in prior 4 weeks^c^	25/66 (37.9)	6/66 (9.1)	6.87 (2.20–29.21)	0.0001
Any antibiotic use in prior 4–12 weeks^c^	10/66 (15.2)	6/66 (9.1)	3.32 (0.85–15.85)	0.09
Azithromycin	2/68 (2.9)	1/67 (1.5)	2.00 (0.01–117.99)	1.00
*β*-lactam and/or *β*-lactamase inhibitor combination	14/68 (20.6)	9/67 (13.4)	1.71 (0.62–5.14)	0.36
Cephalosporin	14/68 (20.6)	1/67 (1.5)	14.00 (2.13–591.97)	0.001
Clindamycin	1/68 (1.5)	0/67 (0.0)	1.00 (0.05–undef)	1.00
Erythromycin/sulfamethoxazole	1/68 (1.5)	0/67 (0.0)	1.00 (0.05–undef)	1.00
Fluoroquinolone	1/68 (1.5)	2/67 (3.0)	0.50 (0.01–9.60)	1.00
Metronidazole	2/68 (2.9)	0/67 (0.0)	2.41 (0.29–undef)	0.50
Trimethoprim/sulfamethoxazole	1/68 (1.5)	0/67 (0.0)	1.00 (0.05–undef)	1.00
Vancomycin (intravenous)	1/68 (1.5)	0/67 (0.0)	1.00 (0.05–undef)	1.00
Any acid reducing medication	4/66 (6.1)	2/67 (3.0)	3.00 (0.24–157.49)	0.63
Any antidepressant	1/67 (1.5)	0/66 (0.0)	1.00 (0.05–undef)	1.00

CI, confidence interval; NICU, neonatal intensive care unit.

Participants could have declined to answer a question; any missing response to a variable was excluded from the denominator.

a Exposure period was during the 12 weeks preceding illness onset.

b The reference group for the higher- and lower-risk exposure categories were participants with no outpatient exposure. A higher-risk outpatient exposure was defined as exposure to an emergency room, outpatient procedure centre, haemodialysis facility, hospital-based outpatient setting, urgent care or ambulatory surgical centre. A lower-risk outpatient exposure was defined as exposure to a dental office, doctor's office, outpatient laboratory or physical therapy centre.

c Two case-patients and one control subject could not recall the exact time-frame of antibiotic exposure during the preceding 12 weeks.

The majority of case-patients (81.8%) and controls (73.5%) reported recent exposure to an outpatient setting, with doctor's office being the most frequently reported setting (Table 2). Higher-risk outpatient exposures were more common among case-patients compared with controls (34.9% *vs.* 17.7%; *P* = 0.03), with emergency room being the most common high-risk exposure in case-patients (18.5% *vs.* 7.4% of controls; *P* = 0.04). Although not statistically significant, a higher percentage of case-patients than controls reported exposures to hospital-based outpatient settings (9.1% *vs.* 1.5%; *P* = 0.06) and outpatient surgery centres (7.7% *vs.* 0.0%; *P* = 0.06).

Antibiotic use in the preceding 12 weeks was reported in 54.4% of case-patients compared with 19.4% of controls (*P* < 0.0001) (Table 2). This difference was most pronounced for exposures to antibiotics in the preceding 4 weeks (37.9% *vs.* 9.1%; *P* = 0.0001) relative to the preceding 4–12 weeks (15.2% *vs.* 9.1%; *P* = 0.09). Overall, the two most commonly reported antibiotic classes were cephalosporins and *β*-lactam and/or *β*-lactamase inhibitor combinations, with cephalosporins being more frequently reported among case-patients than controls (20.6% *vs.* 1.5%, *P* = 0.001). The most commonly reported indication for antibiotic treatment was ear, sinus or respiratory infection among both case-patients (67.6%) and controls (76.9%) (Table 3). Of the 14 case-patients with prior cephalosporin use, eight (57.1%) reported that treatment of an ear, sinus or respiratory infection was the only indication for receiving antibiotics. Exposures to gastric-acid suppressants and antidepressants were also assessed, but no significant differences were found (Table 2).

**Table 3.** Reported indications for antibiotic use among study participants

Indications for antibiotic use^a^	Cases (*N* = 37) No. (%)	Controls (*N* = 13) No. (%)
Ear, sinus or upper respiratory tract infection	25 (67.6)	10 (76.9)
Eye infection	3 (8.1)	1 (7.7)
Skin or soft tissue infection (abscess or cellulitis)	2 (5.4)	1 (7.7)
Surgery	4 (10.8)	0 (0.0)
Urinary tract infection	2 (5.4)	0 (0.0)
Urinary tract infection prophylaxis	2 (5.4)	0 (0.0)
Gastrointestinal infection	1 (2.7)	0 (0.0)
Other	4 (10.8)	1 (7.7)
Unknown reason	1 (2.7)	0 (0.0)

a Participants could report more than one indication for antibiotic use.

Nine (13.6%) of the 66 case-patients with data available, compared with 17 (25.0%) of 68 controls, did not have any outpatient healthcare or relevant medication exposures in the preceding 12 weeks (*P* = 0.12). However, all except one of these nine case-patients had ⩾1 community-based exposures: six (66.7%) had attended daycare, three (33.3%) had a household member who volunteered or worked in a healthcare facility, two (22.2%) had a household member who wore diapers and two (22.2%) had a household member with recent diarrhoeal illness.

Overall, a greater percentage of case-patients reported daycare attendance compared with controls, although the difference was not statistically significant (55.2% *vs.* 37.3%; *P* = 0.06) (Table 4). Case-patients were more likely to have a household member who had a recent diarrhoeal illness (41.3% *vs.* 21.5%, *P* = 0.04); notably, 24% of these case-patients compared with none of the controls had a household member with CDI. No significant difference was detected in the proportion of case-patients and controls who had a recently hospitalised household member or a household member who volunteered or worked in a healthcare facility.

**Table 4.** Univariate analysis: select non-healthcare, household and dietary exposures among study participants

Variable	Cases No. (%)	Controls No. (%)	Unadjusted matched odds ratio (95% CI)	*P-*value
Attended daycare	37/67 (55.2)	25/67 (37.3)	2.09 (0.98–4.75)	0.06
Household exposures^a^				
Household member wore diapers	19/68 (27.9)	25/68 (36.8)	0.67 (0.29–1.46)	0.36
Household member attended child or adult daycare	21/68 (30.9)	20/67 (29.9)	1.07 (0.49–2.32)	1.00
Household member had diarrhoea	26/63 (41.3)	14/65 (21.5)	2.50 (1.05–6.56)	0.04
Household member had *C. difficile* infection	6/25 (24.0)	0/14 (0.0)	1.00 (0.05–undef)	1.00
Household member with overnight stay in a hospital	6/68 (8.8)	3/67 (4.5)	2.00 (0.43–12.36)	0.51
Household member volunteered or worked in a healthcare facility	9/68 (13.2)	16/68 (23.5)	0.50 (0.17–1.32)	0.19
Dietary exposures^b^				
Eggs	11/67 (16.4)	8/67 (11.9)	1.5 (0.48–5.12)	0.61
Dairy	50/67 (74.6)	59/68 (88.1)	0.36 (0.10–1.05)	0.06
Fresh raw vegetables	19/67 (28.4)	19/67 (28.4)	1.00 (0.44–2.26)	1.00
Plant-based protein	12/67 (17.9)	5/67 (7.5)	2.75 (0.81–11.84)	0.12
Red meat	3/67 (4.5)	6/67 (9.0)	0.50 (0.08–2.34)	0.51
Poultry	14/67 (20.9)	13/67 (19.4)	1.10 (0.42–2.89)	1.00
Seafood	1/67 (1.5)	2/67 (3.0)	0.50 (0.01–9.60)	1.00
Diverse diet^c^	18/68 (26.5)	23/68 (33.8)	0.69 (0.29–1.58)	0.44
Well or spring water^d^	59/62 (95.2)	60/66 (90.9)	2.50 (0.41–26.25)	0.45
Formula-fed at least 75% of the time in first 6 months of life	23/68 (33.8)	23/67 (34.3)	0.94 (0.43–2.03)	1.00
Formula-fed almost 100% of the time in first 6 months of life	19/68 (27.9)	15/67 (22.4)	1.30 (0.53–3.31)	0.68

CI, confidence interval.

Participants could have declined to answer a question; any missing response to a variable was excluded from the denominator.

a Exposure period was during the 12 weeks preceding illness onset.

b Unless otherwise specified, dietary exposure is defined as the consumption of a food product with a frequency of more than five times during a typical week.

c Defined as the consumption of any of the food product listed in the table (except for plant-based protein) during a typical week, regardless of the frequency of consumption.

d Source of drinking water around the time of illness onset.

Case-patients were not more likely than controls to be exposed to a diverse diet or to specific food types or water source (Table 4). Furthermore, no difference was detected in the frequency of formula feeding within the first 6 months of life. However, there was less frequent dairy intake in case-patients compared with controls, although this difference was not statistically significant (74.6% *vs.* 88.1%, *P* = 0.06).

In multivariable analysis, only antibiotic exposure in the preceding 12 weeks was significantly associated with CA-CDI (adjusted matched odds ratio 6.25; 95% CI 2.18–17.96). We performed three separate sensitivity analyses: excluding case-patients (and corresponding controls) in the 12–23 month age group, excluding case-patients (and their corresponding controls) who tested positive for another enteric pathogen or who were not tested at all for other enteric pathogens, and excluding case-patients (and their corresponding controls) who were not treated for CDI or had missing treatment information. In all three sensitivity analyses, recent antibiotic exposure remained the only significant finding in multivariable analysis.

## Discussion

This is one of the few multi-site studies to date to explore a wide range of healthcare- and community-related risk factors for CA-CDI in young US children. We found that the majority of case-patients had prior outpatient healthcare exposures (81.8%) and antibiotic use (54.4%), whereas 13.6% did not have any traditional risk factors. Recent antibiotic use was the only independent risk factor for CA-CDI in this study. Although not significant in multivariable analysis, both underlying chronic medical conditions and exposures to high-risk outpatient healthcare settings were more common among case-patients than controls. No community-related risk factors were found to be independently associated with CA-CDI.

Our finding of recent antibiotic use as a risk factor for CA-CDI in children is consistent with previous studies and underscores the importance of outpatient antibiotic stewardship [18, 19, 22]. Not surprisingly, the most frequently reported indication for antibiotic use in this study of young children was ear, sinus or respiratory infection. Inappropriate antibiotic prescribing for acute respiratory tract infections is well-documented, including the overuse of antibiotics not generally recommended for first-line therapy, such as cephalosporins and fluoroquinolones [23–25]. Both of these antibiotic classes have been linked to CA-CDI in children and adults [18, 19, 21, 26]. We observed a significantly higher frequency of cephalosporin use among case-patients, more than half of whom received the antibiotic exclusively for treatment of an ear, sinus or respiratory infection, according to their caregiver. Continued efforts to identify effective interventions to improve outpatient prescribing, particularly for acute respiratory tract infections, are greatly needed.

Although antibiotic use can have long-term impacts on the intestinal microbiota, some studies have found the risk for CDI is highest during and within the first month following antibiotic use [19, 26, 27], including one paediatric study that found a significant association between CA-CDI and antibiotic use only in the prior 30 days [19]. Similarly, when we assessed whether certain time intervals during the 12-week exposure period were associated with higher CA-CDI risk, we found a stronger association with antibiotic use in the prior 4 weeks than in the prior 4–12 weeks.

In univariate analysis, we found case-patients (33.3%) were more likely to have an underlying medical condition, which could lead to more frequent and prolonged outpatient healthcare exposures and potentially more antibiotic exposures, increasing the risk for CDI. Both neurologic and gastrointestinal conditions were the most commonly reported comorbidities in our study, whereas malignancy was the most prevalent condition found in hospitalised children with CDI who were aged 1–5 years [4]. Other studies that included older children with CA-CDI have found as high as 62–73% had underlying comorbidities [19, 28]. We did not identify any specific medical condition or use of a gastrointestinal feeding tube to be associated with CA-CDI, but a history of solid organ transplantation has been associated with CDI in hospitalised children [22], and the presence of a gastrointestinal feeding tube has previously been identified to be a risk factor for CDI among both hospitalised children and those with community-associated disease [19, 22].

We found case-patients were also more likely to have been admitted to the NICU in early infancy. Prior NICU stay could conceivably have affected the course of intestinal maturation and composition as a result of early exposures to hospital organisms or antibiotics [29]. In fact, increased *C. difficile* colonisation has been observed in both preterm infants and infants hospitalised after birth [30]. Interestingly, 61% of our case-patients who had a NICU stay during infancy were diagnosed with CDI during their second year of life. Whether a portion of these case-patients initially acquired their *C. difficile* during their NICU stay and subsequently developed disease is unknown.

Consistent with previous adult and paediatric studies [18, 19, 21], we found in univariate analysis that a higher proportion of case-patients had prior outpatient healthcare exposures. When stratified by types of outpatient exposures, case-patients were more likely to have been exposed to higher-risk settings, such as emergency departments, outpatient procedure centres and hospital-based outpatient clinics. These are settings where there is potentially higher frequency of patient contact with healthcare providers and the environment, which could facilitate the spread of *C. difficile*. In an adult *C. difficile* study, recent care at one or more of these outpatient settings was more common in case-patients than in controls, with recent exposure to an emergency department being an independent risk factor for CA-CDI [21].

Of note, 13.6% of case-patients did not report any recent outpatient healthcare or antibiotic exposures. The majority of these case-patients, however, had recent daycare or relevant household exposures, such as having a household member with recent diarrhoeal illness (including CDI). In another paediatric study, recent exposure to a household member with CDI was a significant risk factor for CA-CDI [18]. Studies that have examined *C. difficile* carriage in households of CDI cases have recovered *C. difficile* from as high as 11–13% of household contacts and 27–33% of domestic pets [31, 32]. *C. difficile* has also been isolated from household environments of persons with CDI [31, 33]. To minimise potential *C. difficile* spread in households, continued education about the importance of hand hygiene is needed, particularly in young children where hand hygiene adherence might be suboptimal, and for caregivers who change diapers. Additional measures, including using separate bathrooms and improving household environmental cleaning and disinfection, especially of the bathroom and diaper changing areas, should be emphasised [34].

The major strengths of this study included enrolment of participants from diverse geographical locations and the use of in-depth interviews to identify exposures that would be missed if relying only on medical records or claims data. The primary limitation of the study was that we could not exclude the possibility that some case-patients were actually colonised with *C. difficile* and had diarrhoea due to another aetiology. This includes patients who tested positive only by a molecular assay as well as patients who tested positive for another enteric pathogen. Information regarding the presence of other enteric pathogens was not available for 10 case-patients. However, among the 58 case-patients who were tested for other enteric pathogens, the majority (82.8%) did not have a positive test for another pathogen, suggesting they likely had true infection with *C. difficile*. It is possible though that a positive test result for another pathogen was not included in the medical record for review, since access to outpatient records was sometimes limited. We believe this may have happened in four instances where the case-patient reported having tested positive for another enteric pathogen, but there was no documentation of the positive test result in the medical records that were available for review. In addition, the majority of case-patients were in the 12–23 month age group, which might include more colonisation than true infection compared with other age groups. However, among case-patients with treatment information available, 80.6% were treated specifically for CDI, suggesting that providers thought most of the case-patients had a true infection. To address the concern that some case-patients might have been colonised, we performed three separate sensitivity analyses (excluding the youngest age group, excluding patients who tested positive for another enteric pathogen or who were not tested at all for other enteric pathogens, and excluding patients who did not receive CDI treatment or had missing treatment information) and still found the same result in multivariable analysis.

This study had additional limitations. Our findings might not be generalisable to older children, who might have different underlying comorbidities and exposure history. Caregivers of study subjects could have been interviewed up to 6 months from the last exposure, which could hinder accurate recall. Recall bias could have occurred given that caregivers of cases may be more likely than caregivers of controls to remember healthcare visits and medication exposures near the time of illness. In addition, pharmacy records were not able to be used to confirm medication exposures. We also required that controls could not have had diarrhoea in the preceding 12 weeks, but we did not apply the same requirement to case-patients; this could have led to more healthcare contact among case-patients if they had other diarrhoeal episodes during the 12-week period. The requirement of participant interviews could have led to the enrolment of more children who were less likely to attend daycare, but efforts were made to call during non-working hours to minimise the possibility of a selection bias. There were an additional 36 eligible case-patients who were not included in the study because they could not be matched to a control. A larger percentage of these 36 case-patients were aged 24–60 months compared with those who were included in the study (83% *vs.* 31%; *P* < 0.0001). The exclusion of these case-patients reduced our sample size of children in the 24–60 month age group, which may have limited our ability to identify risk factors that are more relevant to this age group. Lastly, the small sample size of the overall study may have also limited our ability to identify additional risk factors in multivariable analysis.

In conclusion, antibiotic use is a primary risk factor for CA-CDI in young children. Decreasing unnecessary outpatient antibiotic prescribing, particularly for acute respiratory tract infections, might reduce CA-CDI in this population. Further investigation of other potential risk factors, including outpatient healthcare and household exposures, is needed.
